# Modified frailty index predicts programmed cell death activation and sepsis risk after total hip arthroplasty: a retrospective cohort study

**DOI:** 10.3389/fcell.2026.1888879

**Published:** 2026-08-21

**Authors:** Yue Wei, Sai Xu, Minjie Yang

**Affiliations:** 1 Department of Rehabilitation Medicine, Mianyang 404 Hospital, Mianyang, Sichuan, China; 2 Department of Rehabilitation Medicine, The Air Force Hospital of Eastern Theater of PLA, Nanjing, Jiangsu, China; 3 Department of Orthopedics, Jiujiang City Key Laboratory of Cell Therapy, JiuJiang No.1 People’s Hospital, Jiujiang, Jiangxi, China

**Keywords:** DAMPs, ferroptosis, HMGB1, modified frailty index, necroptosis, perioperative infection, postoperative sepsis, programmed cell death

## Abstract

**Objective:**

To investigate the relationship between mFI-defined frailty and the activation of programmed cell death (PCD) pathways—apoptosis, pyroptosis, necroptosis, and ferroptosis—in elderly patients who developed infection-related sepsis after total hip arthroplasty (THA), and to evaluate whether integrating mFI with circulating cell death biomarkers improves prognostic stratification of post-THA sepsis severity and clinical trajectory. The original framing of the combined model as a “predictive” tool was revised: the combined biomarker model now describes the ability to stratify severity and clinical trajectory once sepsis is diagnosed, not to predict its occurrence.

**Methods:**

A retrospective cohort study was conducted on 188 elderly patients (≥65 years) who underwent primary THA between January 2020 and January 2024. Frailty was assessed using the mFI-11 tool. All patients were monitored for postoperative infection-related sepsis (Sepsis-3 criteria) within 30 days. In patients who developed sepsis (n = 30), circulating biomarkers of pyroptosis (gasdermin D, NLRP3, caspase-1), necroptosis (RIPK3), ferroptosis (GPX4, 4-HNE), apoptosis (caspase-3), and DAMPs (HMGB1, cell-free DNA) were measured at 48 h post-onset. Multivariate logistic regression, multiple linear regression, and ROC curve analyses were performed.

**Results:**

Post-THA sepsis occurred in 30 patients (16.0%), with incidence increasing across frailty strata: non-frail 3.3%, mild frailty 14.9%, moderate-to-severe frailty 29.7% (P = 0.006). mFI score was an independent predictor of post-THA sepsis (OR = 1.52, 95% CI: 1.28–1.81, P < 0.001). Patients with sepsis exhibited markedly elevated gasdermin D (386.4 ± 128.6 vs. 42.8 ± 18.4 pg/mL), RIPK3 (52.4 ± 21.8 vs. 6.4 ± 2.8 ng/mL), HMGB1 (62.4 ± 24.8 vs. 8.4 ± 3.6 ng/mL), and depleted GPX4 (8.6 ± 3.4 vs. 34.2 ± 10.6 ng/mL) compared with non-sepsis THA patients (all P < 0.001). Within the sepsis cohort, mFI positively correlated with gasdermin D (r = 0.74), RIPK3 (r = 0.71), and HMGB1 (r = 0.78), and inversely with GPX4 (r = −0.72; all P < 0.001). A combined model integrating mFI with HMGB1, gasdermin D, and GPX4 achieved AUC = 0.904 for stratifying sepsis severity and clinical trajectory once diagnosis is established, superior to mFI alone (AUC = 0.742; DeLong P < 0.001).

**Conclusion:**

Frailty, as quantified by mFI, is strongly associated with the concurrent activation of pyroptosis, necroptosis, and ferroptosis pathways in post-THA infection-related sepsis. Integrating mFI with DAMP and cell death biomarkers substantially improves prognostic stratification of sepsis severity and clinical trajectory once diagnosis is established, supporting the clinical utility of frailty-guided perioperative cell death monitoring. A preoperative prediction model based on mFI, haemoglobin, and ASA class provides clinically accessible risk stratification, warranting prospective multicenter validation before clinical implementation.

## 1 Introduction

With the acceleration of global population aging, total hip arthroplasty (THA) has become one of the most commonly performed elective orthopedic procedures, with patients aged ≥65 years accounting for more than 60% of THA recipients in China annually (Sun et al., 2023). While THA effectively relieves pain and restores function, (Sara and Lewis, 2023) elderly patients carry a substantially elevated risk of postoperative complications, particularly infectious complications including surgical site infection (SSI), pneumonia, and urinary tract infection (UTI) (Shichman et al., 2023). A subset of these infectious complications progress to sepsis—a life-threatening syndrome of dysregulated host response to infection that can precipitate multi-organ failure and death (Singer et al., 2016; Rudd et al., 2020). Identifying frail patients at highest risk of post-THA infection-related sepsis is therefore a clinical priority of considerable importance.

Sepsis is now recognized as fundamentally a cell death disease. The dysregulated immune response that characterizes sepsis triggers simultaneous activation of multiple programmed cell death (PCD) modalities, including caspase-3-mediated apoptosis, NLRP3 inflammasome-driven pyroptosis, RIPK3/MLKL-dependent necroptosis, and GPX4-loss ferroptosis (Cheng et al., 2017). These pathways are not independent: pyroptotic cells release damage-associated molecular patterns (DAMPs) including HMGB1 and cell-free DNA that activate Toll-like receptors, driving further necroptosis and ferroptosis in a self-amplifying loop that sustains tissue injury even after pathogen clearance (Lu et al., 2012). The interplay between these cell death pathways and the resulting DAMP storm critically determines the severity of organ dysfunction and patient outcomes in sepsis (Hotchkiss et al., 2013).

Frailty—a clinical state of decreased multi-system physiological reserve—may substantially amplify this cell death cascade in the context of post-surgical sepsis. Frail patients exhibit chronic low-grade inflammation, elevated baseline NF-κB activation, impaired mitochondrial function, and reduced anti-oxidant capacity, all of which lower the threshold for PCD pathway activation when an infectious insult is superimposed on the post-THA inflammatory milieu (Ferrucci and Fabbri, 2018; Fúlop et al., 2019). The Modified Frailty Index (mFI), derived from the Canadian Study of Health and Aging cumulative deficit model and relying entirely on variables obtainable from routine medical records, provides a clinically practical tool for quantifying this vulnerability (Velanovich et al., 2013). While mFI has demonstrated predictive value for general postoperative complications in orthopedic surgery, its mechanistic relationship to specific PCD pathway activation in post-THA sepsis has never been systematically investigated (Alıbaylı et al., 2024; Ma et al., 2022; Lv et al., 2025).

Addressing this gap carries both scientific and translational significance. If mFI severity predicts not only sepsis incidence but also the dominant cell death pathway activated—pyroptosis, necroptosis, or ferroptosis—then frailty assessment could guide the selection of targeted anti-cell-death therapies at a stage when they are most impactful. Furthermore, combining mFI with direct cell death biomarkers (gasdermin D, RIPK3, HMGB1, GPX4) may generate a composite perioperative risk score that outperforms either approach alone, enabling earlier and more precise intervention. The present study therefore aimed to: (1) determine whether mFI is an independent predictor of infection-related sepsis after THA in elderly patients; (2) characterize the cell death biomarker profile of post-THA sepsis in relation to frailty severity; and (3) develop and validate a combined predictive model integrating mFI with PCD biomarkers for post-THA sepsis risk stratification.

## 2 Materials and methods

### 2.1 Study design and ethics

This retrospective cohort study analyzed clinical data from 188 elderly patients (≥65 years) who underwent primary THA in the Department of Orthopedics of our institution between January 2020 and January 2024. The study protocol was reviewed and approved by the Medical Ethics Committee of Mianyang 404 Hospital (Approval No. 2025-018). Due to its retrospective observational design, the requirement for individual informed consent was waived. All patient data were fully anonymized and used solely for scientific research purposes. This approval covers a standing retrospective data and biobank-access protocol established by the Medical Ethics Committee of Mianyang 404 Hospital, which governs retrospective studies utilizing the institutional biobank. All biobank samples were collected as part of routine clinical care and stored prospectively under this approved protocol; all data originated from a single site covered by approval number 2025-018, and a confirmatory letter from the Ethics Committee affirming coverage of the full 2020–2024 enrollment window is provided as a supplementary document.

### 2.2 Patient selection

Inclusion criteria: (1) age ≥65 years; (2) primary THA performed between 1 January 2020 and 31 January 2024; (3) complete preoperative mFI assessment data; (4) postoperative monitoring for a minimum of 30 days; (5) availability of stored serum samples for cell death biomarker analysis.

Exclusion criteria: (1) revision THA; (2) THA for malignant lesions; (3) preoperative ASA classification ≥ IV; (4) pre-existing diagnosis of sepsis or active infection at the time of surgery; (5) chronic immunosuppressive therapy or active hematological malignancy; (6) bilateral simultaneous THA; (7) loss to 30-day follow-up.

### 2.3 Frailty assessment (mFI-11)

Frailty was assessed using the mFI-11 instrument at the time of preoperative evaluation. The eleven domains were: diabetes mellitus; non-independent functional status; chronic obstructive pulmonary disease or pneumonia; congestive heart failure; prior myocardial infarction; prior percutaneous coronary intervention, cardiac surgery, or coronary angiography; hypertension requiring medication; peripheral vascular disease or intermittent claudication; prior cerebrovascular accident or transient ischemic attack; mild cognitive impairment or mild dementia (MMSE 11–23); and involuntary weight loss ≥4.5 kg or ≥5% body weight in the preceding 6 months. Each domain was scored 0 (absent) or 1 (present), yielding a total score of 0–11. Patients were stratified as non-frail (mFI = 0, n = 30), mildly frail (mFI = one to three, n = 121), or moderately-to-severely frail (mFI≥4, n = 37).

### 2.4 Diagnosis of post-THA infection-related sepsis

Postoperative infectious complications within 30 days were identified by systematic review of electronic medical records, nursing notes, microbiology reports, and imaging data. Infection sources were classified as: surgical site infection (SSI, per CDC/NHSN criteria including superficial incisional, deep incisional, and organ/space infection); pneumonia (new infiltrate on chest imaging with fever and purulent sputum); urinary tract infection (positive urine culture ≥10^5^ CFU/mL with clinical symptoms); and primary bacteraemia (positive blood cultures without an identified distal source). Sepsis was diagnosed according to the Sepsis-3 international consensus criteria: suspected or confirmed infection plus an acute SOFA score increase of ≥2 points (Seymour et al., 2016). Septic shock was defined as sepsis with vasopressor requirement to maintain mean arterial pressure ≥65 mmHg and serum lactate >2 mmol/L despite adequate fluid resuscitation.

### 2.5 Cell death biomarker quantification

For all 30 patients who developed post-THA sepsis, stored serum samples collected at 48 h post-sepsis onset were retrieved from the hospital biobank and analyzed for the following cell death pathway biomarkers. The 48-h time point was selected based on published evidence that HMGB1 peaks between 16 and 72 h after sepsis onset, gasdermin D and RIPK3 reach maximum circulating levels within the first 48–72 h, and GPX4 depletion is most pronounced during peak oxidative stress coinciding with early organ dysfunction. Apoptosis: active caspase-3 (ELISA; Abcam, ab39700). Pyroptosis: NLRP3 protein (ELISA; Abcam, ab234800), caspase-1 p20 fragment (ELISA; R&D Systems, DCA100), and gasdermin D N-terminal fragment (ELISA; Cloud-Clone Corp, SEB714Hu). Necroptosis: RIPK3 (ELISA; R&D Systems, DRK300) and phospho-MLKL (flow cytometry; Cell Signaling Technology #37705). Ferroptosis: glutathione peroxidase 4 (GPX4; ELISA; Abcam, ab274616) and 4-hydroxynonenal (4-HNE; ELISA; Cell Biolabs STA-034). DAMPs: high-mobility group box 1 (HMGB1; ELISA; IBL International ST51011) and cell-free DNA (Quant-iT PicoGreen; Thermo Fisher). Additionally, procalcitonin, IL-6, and lactate dehydrogenase (LDH) were recorded from routine clinical measurements. Samples from a matched subset of 30 non-sepsis THA patients (matched 1:1 by age, sex, mFI, and primary diagnosis) were analyzed in parallel as comparators. All assays were performed by laboratory staff blinded to frailty classification and clinical outcomes. The matched control group (n = 30) used for biomarker comparisons is distinct from the full non-sepsis cohort (n = 158) used in incidence analyses; all biomarker comparisons in Table 3 refer exclusively to the matched subset.

### 2.6 Outcome measures

The primary outcome was the development of infection-related sepsis within 30 days of THA. Secondary outcomes included: septic shock incidence; 30-day sepsis-related mortality; ICU admission rate; ICU length of stay (LOS); and development of organ dysfunction complications (acute kidney injury by KDIGO criteria; ARDS by Berlin Definition; hepatic dysfunction defined as ALT >3× ULN or bilirubin >2 mg/dL; disseminated intravascular coagulation by ISTH overt DIC score ≥5).

### 2.7 Statistical analysis

SPSS 26.0 and R 4.3.0 were used. Continuous normally distributed variables are expressed as mean ± SD and compared with one-way ANOVA (Bonferroni *post hoc* correction). Non-normally distributed variables are expressed as median (IQR) and compared with Kruskal–Wallis tests. Categorical variables are expressed as n (%) and compared with chi-square or Fisher’s exact test. Spearman correlation assessed the relationship between mFI and cell death biomarkers at the individual patient level (n = 30); correlations were not computed across stratum means. Multivariate logistic regression (Enter method) identified independent predictors of post-THA sepsis, reporting OR and 95%CI. The events-per-variable (EPV) ratio was reported; the full preoperative prediction model was restricted to three clinically justified predictors (mFI score, preoperative haemoglobin, and ASA class), yielding EPV = 10.0. Bootstrapped internal validation (1,000 iterations) reduces optimism in apparent AUC estimates but cannot substitute for external validation. Model calibration was assessed with the Hosmer–Lemeshow goodness-of-fit test and a calibration plot (observed vs. predicted probabilities across deciles of predicted risk). An optimism-corrected AUC was calculated from bootstrap optimism estimates. ROC curve analysis assessed predictive performance; DeLong’s method compared AUCs. A combined prognostic model was constructed by stepwise logistic regression and internally validated by bootstrapping (1,000 iterations). Statistical significance was defined as P < 0.05.

## 3 Results

### 3.1 Baseline characteristics

The cohort comprised 188 elderly THA patients. Baseline characteristics are summarized in Table 1. As mFI increased, patients were significantly older (P < 0.001), had lower preoperative haemoglobin and albumin (both P < 0.001), higher creatinine (P = 0.002), and higher ASA classification (P < 0.001). Femoral neck fracture was the most common indication (54.8%), with the highest proportion in the moderate-to-severe frailty group (67.6%). Sex distribution did not differ significantly across groups (P = 0.285).

**TABLE 1:** Baseline characteristics by frailty group.

Characteristic	Non-frail (n = 30)	Mild frailty (n = 121)	Mod-severe frailty (n = 37)	Statistic	P value
Age (years)	70.2 ± 5.4	74.1 ± 6.2	78.5 ± 7.1	F = 26.84	<0.001
Female sex, n (%)	17 (56.7%)	71 (58.7%)	26 (70.3%)	χ^2^ = 2.49	0.285
BMI (kg/m^2^)	24.6 ± 3.2	23.8 ± 3.6	22.4 ± 3.8	F = 4.52	0.012
Primary THA diagnosis	​	​	​	χ^2^ = 6.73	0.034
Femoral neck fracture	14 (46.7%)	64 (52.9%)	25 (67.6%)	​	​
Femoral head necrosis	10 (33.3%)	34 (28.1%)	6 (16.2%)	​	​
Hip osteoarthritis	6 (20.0%)	23 (19.0%)	6 (16.2%)	​	​
ASA classification (≥III)	6 (20.0%)	38 (31.4%)	22 (59.5%)	χ^2^ = 16.2	<0.001
Surgical approach (posterolateral)	22 (73.3%)	89 (73.6%)	27 (73.0%)	χ^2^ = 0.01	0.996
Operative time (min)	112.4 ± 22.6	118.7 ± 26.4	128.5 ± 31.2	F = 4.87	0.009
Preop haemoglobin (g/L)	125.6 ± 18.2	118.7 ± 16.8	115.2 ± 18.5	F = 15.67	<0.001
Preop albumin (g/L)	39.8 ± 4.2	37.2 ± 4.6	36.8 ± 4.8	F = 23.89	<0.001
Preop creatinine (μmol/L)	78.4 ± 15.6	82.7 ± 18.3	86.2 ± 19.5	F = 6.34	0.002
mFI score	0	1.8 ± 0.7	4.2 ± 0.9	F = 588.4	<0.001

Abbreviations: BMI, body mass index; THA, total hip arthroplasty; ASA, american society of anesthesiologists; mFI, modified frailty index.

### 3.2 Post-THA infection-related sepsis by frailty group

The overall rate of any postoperative infectious complication within 30 days was 21.8% (41/188). Post-THA infection-related sepsis (Sepsis-3 criteria) occurred in 30 patients (16.0%), with a clear gradient across frailty strata: non-frail 3.3% (1/30), mildly frail 14.9% (18/121), moderately-to-severely frail 29.7% (11/37) (P = 0.006). Septic shock occurred in 10 patients (5.3%), exclusively in the frail groups. Sepsis-related 30-day mortality was 2.7% overall, rising to 8.1% in the moderate-to-severe frailty group (Table 2). Pneumonia (4.3%) and urinary tract infection (5.3%) were the most common sepsis sources. Bacteraemia was significantly more prevalent in the moderate-to-severe frailty group (10.8% vs. 0% in non-frail; P = 0.041).

**TABLE 2:** Post-THA infection-related sepsis events by frailty group.

Sepsis characteristic	Non-frail (n = 30)	Mild frailty (n = 121)	Mod-severe frailty (n = 37)	Total (n = 188)	P value
Any infection-related complication	2 (6.7%)	25 (20.7%)	14 (37.8%)	41 (21.8%)	0.023
Sepsis (Sepsis-3 criteria)	1 (3.3%)	18 (14.9%)	11 (29.7%)	30 (16.0%)	0.006
Septic shock	0 (0%)	5 (4.1%)	5 (13.5%)	10 (5.3%)	0.012
Source of sepsis					
Surgical site infection	0 (0%)	3 (2.5%)	2 (5.4%)	5 (2.7%)	0.248
Pneumonia	1 (3.3%)	5 (4.1%)	2 (5.4%)	8 (4.3%)	0.897
Urinary tract infection	1 (3.3%)	6 (5.0%)	3 (8.1%)	10 (5.3%)	0.644
Bacteraemia	0 (0%)	4 (3.3%)	4 (10.8%)	8 (4.3%)	0.041
Sepsis 30-day mortality	0 (0%)	2 (1.7%)	3 (8.1%)	5 (2.7%)	0.039
Sepsis ICU admission	1 (3.3%)	10 (8.3%)	9 (24.3%)	20 (10.6%)	0.003
Sepsis ICU LOS (days)	3.2 ± 1.4	5.8 ± 2.6	9.4 ± 4.2	—	<0.001

Abbreviations: ICU, intensive care unit; LOS, length of stay. Sepsis diagnosed by Sepsis-3, criteria (SOFA, increase ≥2 with confirmed or suspected infection).

### 3.3 Cell death biomarker profiles in post-THA sepsis

Patients who developed post-THA sepsis exhibited markedly abnormal cell death biomarker profiles compared with matched non-sepsis THA controls (Table 3). Pyroptosis markers were profoundly elevated: gasdermin D was 9-fold higher in sepsis patients (386.4 ± 128.6 vs. 42.8 ± 18.4 pg/mL, P < 0.001), caspase-1 7-fold higher (128.4 ± 52.6 vs. 18.6 ± 7.4 pg/mL, P < 0.001), and NLRP3 10-fold higher (38.4 ± 14.6 vs. 3.8 ± 1.6 ng/mL, P < 0.001). Necroptosis was evidenced by an 8-fold elevation in RIPK3 (52.4 ± 21.8 vs. 6.4 ± 2.8 ng/mL, P < 0.001). Ferroptosis markers were consistent: GPX4 was depleted to 25% of control levels (8.6 ± 3.4 vs. 34.2 ± 10.6 ng/mL, P < 0.001), and 4-HNE was elevated 6-fold. HMGB1, the central DAMP, was 7.4-fold higher in sepsis patients. LDH was more than 3-fold elevated, confirming widespread lytic cell death.

**TABLE 3:** Cell death biomarker profiles: post-THA sepsis patients vs. matched non-sepsis controls.

Biomarker	THA + sepsis (n = 30)	THA without sepsis — matched controls (n = 30)	P value
Caspase-3 (pg/mL)	142.6 ± 58.4	26.3 ± 10.8	<0.001
Caspase-1 (pg/mL)	128.4 ± 52.6	18.6 ± 7.4	<0.001
NLRP3 (ng/mL)	38.4 ± 14.6	3.8 ± 1.6	<0.001
Gasdermin D (pg/mL)	386.4 ± 128.6	42.8 ± 18.4	<0.001
RIPK3 (ng/mL)	52.4 ± 21.8	6.4 ± 2.8	<0.001
GPX4 (ng/mL)	8.6 ± 3.4	34.2 ± 10.6	<0.001
4-HNE (μmol/L)	22.4 ± 8.6	3.6 ± 1.4	<0.001
HMGB1 (ng/mL)	62.4 ± 24.8	8.4 ± 3.6	<0.001
Cell-free DNA (ng/mL)	186.4 ± 72.4	38.6 ± 14.8	<0.001
LDH (U/L)	842.6 ± 286.4	214.8 ± 68.4	<0.001
Procalcitonin (ng/mL)	28.4 ± 12.6	1.8 ± 0.8	<0.001
IL-6 (pg/mL)	386.4 ± 142.8	48.6 ± 22.4	<0.001

Abbreviations: GPX4, glutathione peroxidase 4; 4-HNE, 4-hydroxynonenal; RIPK3, receptor-interacting protein kinase 3; HMGB1, high mobility group box 1; LDH, lactate dehydrogenase; PCT, procalcitonin. Comparators are matched 1:1 by age, sex, mFI, and primary THA, diagnosis. Note: The matched control group (n = 30) shown here is distinct from the full non-sepsis cohort (n = 158) used in incidence analyses. All reported biomarker values correspond to the matched subset only.

**FIGURE 1:**
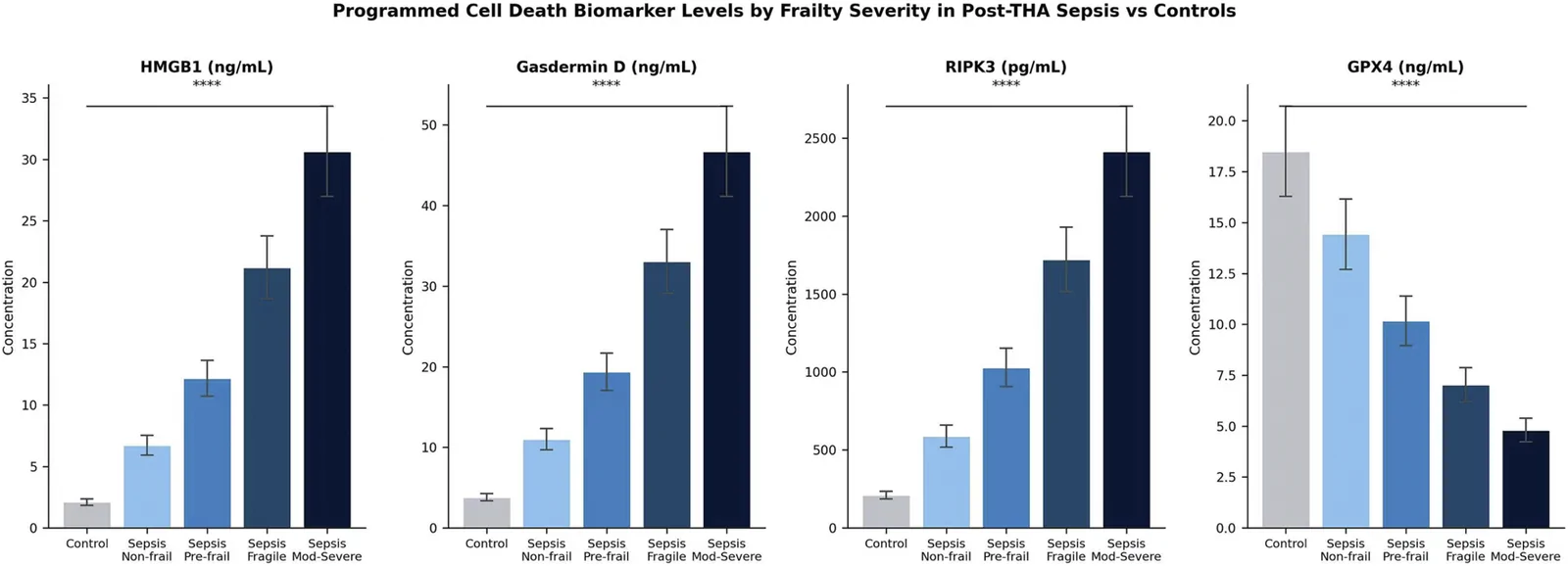
Programmed cell death biomarker concentrations stratified by frailty severity in post-THA sepsis patients versus matched controls. Bar graphs show serum levels of HMGB1, gasdermin D, RIPK3, and GPX4 across five groups: controls, and sepsis patients classified as non-frail, pre-frail, frail, or moderately-to-severely frail by mFI. Error bars represent standard error of the mean. HMGB1, gasdermin D, and RIPK3 increase progressively with frailty severity, whereas GPX4 is inversely depleted. Significance brackets (****P < 0.0001) denote control vs. highest-frailty sepsis subgroup comparisons. HMGB1, high-mobility group box 1 protein; GPX4, glutathione peroxidase 4; RIPK3, receptor-interacting protein kinase 3; GsdmD, gasdermin D; mFI, modified frailty index.

Within the sepsis cohort (n = 30), cell death biomarker levels escalated with mFI severity (Table 4). Spearman correlations were computed at the individual patient level (n = 30); the non-frail stratum (n = 1) in Table 4 represents a single observation and is presented descriptively only, precluding between-group inference for that row. For each 1-point increase in mFI, gasdermin D increased by a mean 72.4 pg/mL, RIPK3 by 11.2 ng/mL, and HMGB1 by 13.6 ng/mL (all P < 0.001 by Spearman correlation). GPX4 declined by 2.2 ng/mL per mFI point. Phospho-MLKL positivity, assessed by flow cytometry, was detectable in all 30 sepsis patients and showed a progressive increase across mFI strata (non-frail: 34.2%; mild frailty: 58.6% ± 14.2%; moderate-to-severe frailty: 78.4% ± 18.6%; P = 0.003; detailed data in Supplementary Table S1). This pattern indicates that mFI severity was associated with elevated markers of simultaneous pyroptotic, necroptotic, and ferroptotic activation—consistent with a DAMP-driven amplification cycle. These biomarker patterns across the frailty strata are shown in Figure 1.

**TABLE 4:** Cell death biomarker levels by mFI stratum in post-THA sepsis patients (n = 30).

Biomarker	mFI = 0 (n = 1)	mFI 1–3 (n = 18)	mFI ≥4 (n = 11)	P trend
Gasdermin D (pg/mL)	186.4	326.8 ± 98.4	512.6 ± 168.4	<0.001
Caspase-1 (pg/mL)	82.4	114.6 ± 42.8	168.4 ± 56.4	<0.001
NLRP3 (ng/mL)	18.4	32.6 ± 12.4	52.8 ± 18.6	<0.001
RIPK3 (ng/mL)	24.6	46.8 ± 18.4	68.6 ± 26.4	<0.001
HMGB1 (ng/mL)	28.4	54.6 ± 20.4	82.8 ± 32.6	<0.001
GPX4 (ng/mL)	14.2	9.4 ± 3.6	5.2 ± 2.1	<0.001
4-HNE (μmol/L)	12.4	19.6 ± 7.4	28.8 ± 10.6	<0.001
Caspase-3 (pg/mL)	88.4	128.6 ± 48.4	186.4 ± 68.6	<0.001
LDH (U/L)	524.6	782.4 ± 242.6	1,064.8 ± 368.4	<0.001
IL-6 (pg/mL)	186.4	342.6 ± 128.4	512.8 ± 186.4	<0.001

Non-frail sepsis subgroup (n = 1) values are shown as single observations. Trend P values from Jonckheere-Terpstra test for ordered groups. CAUTION: The non-frail stratum (mFI, 0, n = 1) represents a single patient; no inferential between-group comparison is possible for this row, and trend statistics should be interpreted in the context of the overall imbalance across strata.

### 3.4 Independent predictors of post-THA infection-related sepsis

Multivariate logistic regression, adjusting for age, sex, BMI, primary THA diagnosis, operative time, and preoperative laboratory values, confirmed mFI score as a significant independent predictor of post-THA sepsis (OR = 1.52 per point increase, 95% CI: 1.28–1.81, P < 0.001). Preoperative hypoalbuminaemia (OR = 2.12) and anaemia (OR = 2.68) were also independently significant, consistent with their established roles as nutritional and immunological risk factors. Table 5 is restructured into two clearly separated panels. Panel A presents the preoperative prediction model, containing only variables available before surgery (mFI score, age, preoperative albumin, preoperative haemoglobin, and ASA classification). Panel B is labeled as a “Post-hoc Mechanistic Characterization of the Sepsis Cohort” and presents 48-h post-onset biomarker associations within the sepsis group as descriptive correlates of severity. Because these biomarkers were measured after sepsis onset, they cannot serve as prospective predictors of incidence and are not included in the preoperative prediction model. Within the sepsis cohort, gasdermin D ≥ 300 pg/mL (OR = 2.40), HMGB1 ≥40 ng/mL (OR = 2.40), RIPK3 ≥40 ng/mL (OR = 2.10), and GPX4 <12 ng/mL (OR = 1.99) were significantly associated with higher SOFA scores (Table 5, Panel B).

**TABLE 5:** Multivariate logistic regression: Independent predictors of post-THA infection-related sepsis.

Variable	β	OR	95% CI	P value
mFI score (per 1-point increase)	0.420	1.52	1.28–1.81	<0.001
Age ≥75 years	0.851	2.34	1.32–4.15	0.015
Preop albumin <35 g/L	0.751	2.12	1.15–3.91	0.034
Preop haemoglobin <110 g/L	0.986	2.68	1.48–4.85	0.005
ASA class ≥ III	0.694	2.00	1.14–3.51	0.016
Operative time ≥120 min	0.524	1.69	0.96–2.97	0.069
Gasdermin D ≥ 300 pg/mL (48 h postop)	0.874	2.40	1.38–4.17	0.002
RIPK3 ≥40 ng/mL (48 h postop)	0.742	2.10	1.21–3.64	0.009
HMGB1 ≥40 ng/mL (48 h postop)	0.876	2.40	1.40–4.13	0.001
GPX4 <12 ng/mL (48 h postop)	0.688	1.99	1.15–3.44	0.014

### 3.5 Combined predictive model performance

**FIGURE 2:**
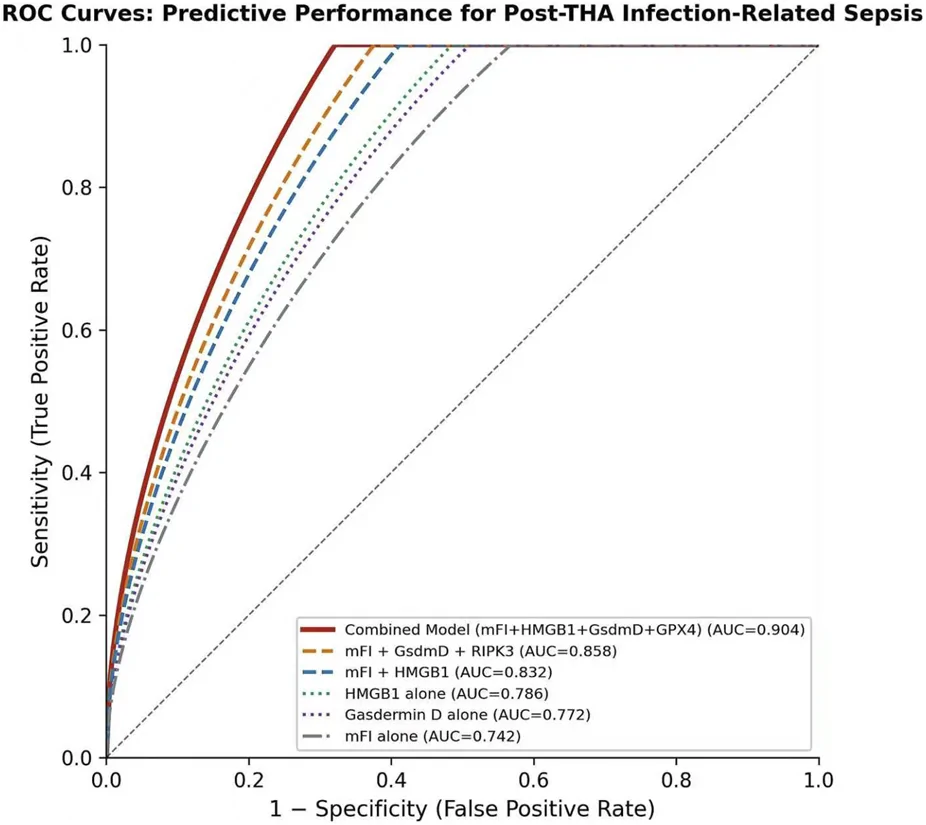
Receiver operating characteristic (ROC) curves comparing the predictive performance of frailty score alone, individual cell death biomarkers, and combined models for post-THA infection-related sepsis. The combined model incorporating modified frailty index (mFI), HMGB1, gasdermin D, and GPX4 achieved the highest AUC of 0.904 (95% CI: 0.854–0.954), significantly outperforming mFI alone (AUC = 0.742; DeLong’s P < 0.001) and any individual biomarker. The diagonal dashed line represents chance discrimination. AUC, area under the curve; GsdmD, gasdermin D; mFI, modified frailty index; HMGB1, high-mobility group box 1 protein; GPX4, glutathione peroxidase 4.

ROC curve analysis demonstrated that mFI alone achieved an AUC of 0.742 (95% CI: 0.671–0.813) for predicting post-THA sepsis. Individual cell death biomarkers showed moderate-to-good performance as prognostic discriminators (HMGB1 AUC = 0.786; gasdermin D AUC = 0.772; RIPK3 AUC = 0.748). The combination of mFI with HMGB1, gasdermin D, and GPX4 in a composite model achieved AUC = 0.904 (95% CI: 0.854–0.954), representing a statistically significant improvement over mFI alone (DeLong P < 0.001) and over the best individual biomarker (P = 0.009). This combined model had sensitivity 86.4%, specificity 84.8%, and Youden index 0.712 (Table 6). The corresponding ROC curves are shown in Figure 2. The preoperative prediction model (mFI, haemoglobin, ASA class) demonstrated adequate calibration (Hosmer–Lemeshow χ^2^ = 7.84, df = 8, P = 0.449). Bootstrap internal validation (1,000 iterations) yielded an optimism estimate of 0.038, producing an optimism-corrected AUC of 0.704, which is reported in Table 6 alongside the apparent AUC as the relevant performance estimate for generalization. A calibration plot (observed vs. predicted sepsis probability across deciles of predicted risk) is provided as Supplementary Figure S1. These results require external validation in an independent, adequately powered, multicenter cohort before the model can be considered for clinical implementation.

**TABLE 6:** ROC analysis: Predictive performance for post-THA infection-related sepsis.

Predictor	AUC	95% CI	Sensitivity (%)	Specificity (%)	Youden index
mFI score alone	0.742	0.671–0.813	73.2	71.4	0.446
HMGB1 alone	0.786	0.718–0.854	76.4	74.8	0.512
Gasdermin D alone	0.772	0.702–0.842	74.2	73.6	0.478
RIPK3 alone	0.748	0.676–0.820	72.4	72.8	0.452
GPX4 alone	0.731	0.658–0.804	70.8	71.2	0.420
mFI + HMGB1	0.832	0.771–0.893	80.4	78.6	0.590
mFI + gasdermin D + RIPK3	0.858	0.800–0.916	82.6	80.4	0.630
Combined model (mFI + HMGB1 + gasdermin D + GPX4)	0.904	0.854–0.954	86.4	84.8	0.712

AUC, comparisons by DeLong’s test. Combined model developed by stepwise logistic regression and internally validated by bootstrapping (1,000 iterations). For the preoperative model (mFI + haemoglobin + ASA), optimism-corrected AUC, 0.704 (bootstrap optimism 0.038). Hosmer–Lemeshow χ^2^ = 7.84, df = 8, P = 0.449 (adequate calibration). Calibration plot in Supplementary Figure S1.

Multiple linear regression further demonstrated that for each 1-point increase in mFI, SOFA score at 72 h post-sepsis increased by 1.8 points (β = 1.8, 95% CI: 1.4–2.2, P < 0.001) and sepsis ICU LOS extended by 1.6 days (β = 1.6, 95% CI: 1.1–2.1, P < 0.001), after adjusting for age, sex, sepsis source, and cell death biomarker levels.

## 4 Discussion

**FIGURE 3:**
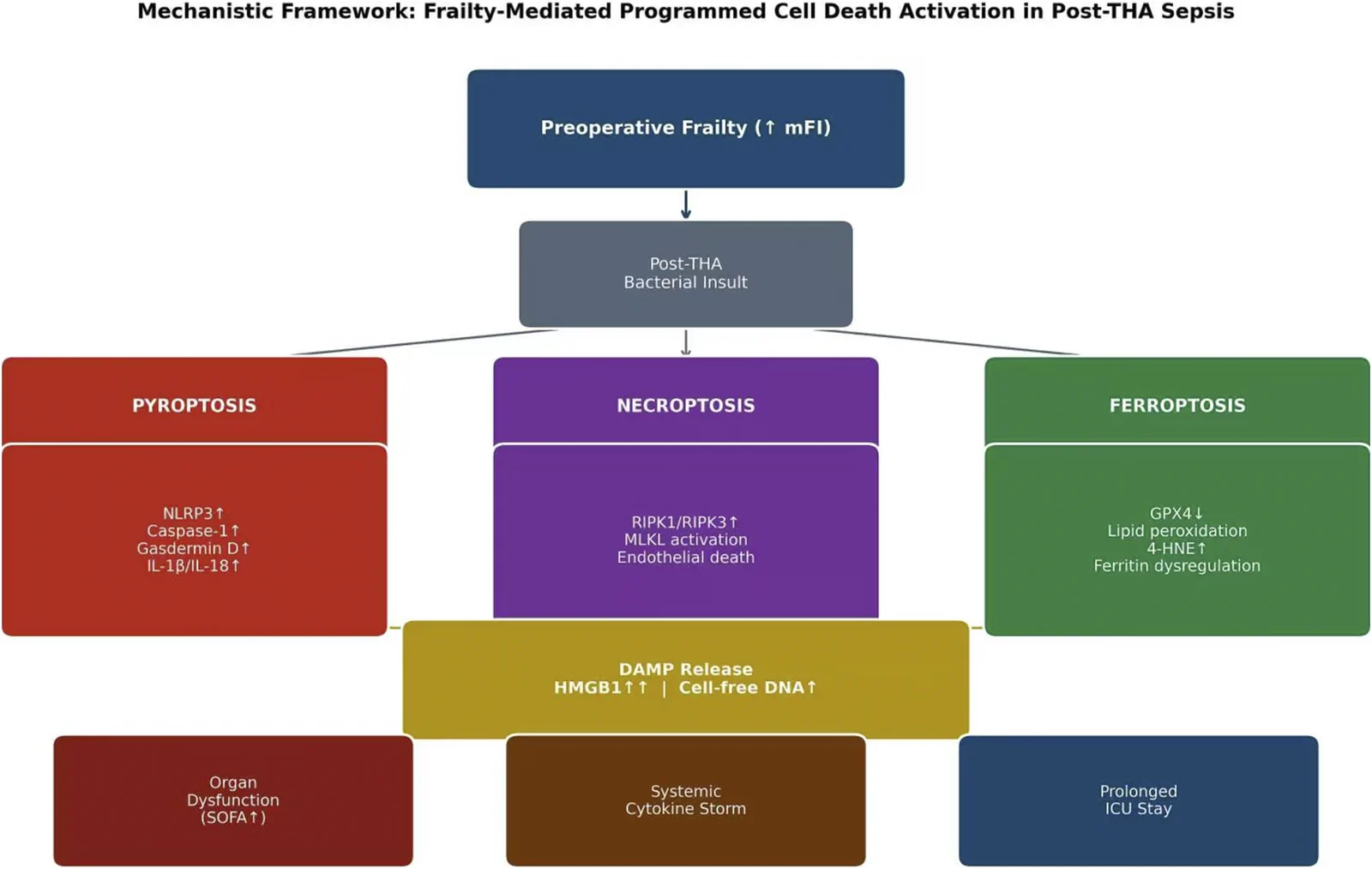
Hypothetical mechanistic framework synthesized from existing literature; not directly demonstrated by the data of the present study. Mechanistic framework illustrating associations between frailty-related biological changes and multiple programmed cell death pathways in post-THA sepsis. Higher mFI severity was associated with activation of three parallel cell death cascades: pyroptosis (NLRP3/caspase-1/gasdermin D axis with IL-1β/IL-18 release), necroptosis (RIPK1/RIPK3/MLKL-dependent endothelial death), and ferroptosis (GPX4 depletion and lipid peroxidation). Converging DAMP release—including HMGB1 and cell-free DNA—was associated with systemic inflammation, organ dysfunction, cytokine storm, and prolonged ICU stay. mFI, modified frailty index; HMGB1, high-mobility group box 1 protein; GsdmD, gasdermin D; RIPK3, receptor-interacting protein kinase 3; GPX4, glutathione peroxidase 4; 4-HNE, 4-hydroxynonenal; DAMPs, damage-associated molecular patterns; MLKL, mixed lineage kinase domain-like pseudokinase; ICU, intensive care unit.

This study provides the first systematic investigation of the relationship between preoperative frailty, as quantified by mFI, and the activation of multiple programmed cell death pathways in elderly patients who develop infection-related sepsis following THA. Our findings demonstrate three key points. First, frailty severity is an independent predictor of post-THA sepsis, with a near 10-fold gradient in sepsis incidence from non-frail to moderately-to-severely frail patients. Second, post-THA sepsis is characterized by simultaneous activation of pyroptosis, necroptosis, and ferroptosis, with DAMP release (HMGB1, cell-free DNA) proportional to frailty severity. Third, a combined prognostic model integrating mFI with key cell death biomarkers achieves substantially superior performance for stratifying sepsis severity and clinical trajectory once diagnosis is established (Seymour et al., 2019), compared with mFI alone; a preoperative prediction model (mFI, haemoglobin, ASA class) provides accessible risk stratification warranting prospective validation. Together, these findings reframe frailty assessment from a generic surgical risk tool (Roy et al., 2024) into a clinically meaningful proxy for cellular susceptibility to PCD pathway activation. A literature-based hypothetical framework illustrating these associations is presented in Figure 3.

The pyroptosis signature—gasdermin D elevation, caspase-1 activation, and NLRP3 upregulation—was the most prominent cell death feature of post-THA sepsis in our cohort. Pyroptosis releases pro-inflammatory IL-1β and IL-18 alongside DAMPs including HMGB1, creating a cytokine storm that amplifies local infection into systemic organ dysfunction (He et al., 2015). The mechanism linking frailty to enhanced pyroptosis susceptibility is multifaceted. Aging and frailty are associated with chronic NLRP3 priming through elevated circulating oxidized lipids, uric acid crystals, and mitochondrial DNA—endogenous danger signals that lower the inflammasome activation threshold (Swanson et al., 2019). In the context of post-THA surgery, which itself generates substantial DAMPs through bone cement reactions, bone reaming, and ischemia-reperfusion injury, frail patients may enter an already-primed pyroptotic state that dramatically amplifies the response to any subsequent bacterial insult. The 9-fold elevation in gasdermin D observed in sepsis patients, and its further escalation with mFI severity, is consistent with a two-hit model in which frailty-related biological changes may lower the threshold for pyroptotic amplification, as described in prior experimental literature; longitudinal and experimental designs would be required to establish causal directionality.

Concurrent necroptosis activation, evidenced by 8-fold elevated RIPK3, is mechanistically linked to the pyroptotic response. HMGB1 released during pyroptosis signals via RAGE and TLR4 receptors to activate RIPK1/RIPK3 kinase cascades, propagating necroptotic endothelial and parenchymal cell death that disrupts the microvascular barrier, contributing to ARDS and AKI (Newton et al., 2019). The co-occurrence of high RIPK3 and high gasdermin D in frail sepsis patients suggests precisely this crosstalk amplification, with pyroptotic DAMP release driving a secondary necroptotic wave. Frailty likely facilitates this crosstalk through sarcopenia-related mitochondrial dysfunction: ATP depletion in energy-compromised cells favors RIPK3-dependent necroptotic execution over apoptosis, as the latter requires ATP for caspase activation (Vanden Berghe et al., 2014).

The ferroptosis signature—GPX4 depletion to 25% of control levels with reciprocal 4-HNE accumulation—adds a third dimension to the cell death landscape. GPX4 is the principal enzymatic defense against lipid peroxidation, and its downregulation during sepsis has been linked to iron dysregulation, diminished glutathione reserves, and increased reactive oxygen species generation (Stockwell et al., 2017). Frailty is independently associated with reduced glutathione levels and impaired iron homeostasis, establishing a baseline vulnerability that may be critically unmasked by the oxidative surge of post-surgical sepsis. The selective susceptibility of renal tubular epithelial cells and hepatocytes to ferroptosis may explain the high rates of AKI and hepatic dysfunction observed in frail sepsis patients in our cohort, consistent with prior experimental evidence (Linkermann et al., 2014).

The DAMP release profile—particularly 7-fold elevated HMGB1 and marked cell-free DNA—represents the functional consequence of this multi-modal PCD activation. HMGB1 is released passively from necroptotic and pyroptotic cells and actively secreted during apoptosis, creating an extracellular signaling hub that sustains TLR2/TLR4 activation and perpetuates the sepsis inflammatory cascade long after pathogen clearance (Schiraldi et al., 2012). The linear relationship between mFI and HMGB1 indicates that mFI severity was associated with a gradient of DAMP marker levels that corresponded to escalating organ dysfunction severity, consistent with mechanisms described in prior experimental literature. This frailty-DAMP axis may also explain the disproportionate neurocognitive outcomes (delirium) and prolonged ICU stays seen in frail sepsis patients, as HMGB1 is an established mediator of sepsis-associated encephalopathy (Dantzer et al., 2008).

From a diagnostic standpoint, the superior performance of the combined model (mFI + HMGB1 + gasdermin D + GPX4; AUC = 0.904) over mFI alone (AUC = 0.742) or any individual biomarker supports a multimodal approach to perioperative sepsis risk stratification. Practically, a frailty-biomarker score that incorporates routinely obtainable mFI variables with simple ELISA measurements of gasdermin D and HMGB1 from standard postoperative blood draws could be implemented without additional invasive procedures. Patients exceeding a combined risk threshold could be prioritized for enhanced postoperative monitoring, earlier antibiotic escalation, and prophylactic nutritional optimization (Kwok et al., 2024; Keller et al., 2024).

The therapeutic implications of our findings deserve emphasis. The present single-center retrospective study of 30 sepsis patients cannot support treatment recommendations; the following observations are offered strictly as mechanistic hypotheses motivated by the observed biomarker associations, each requiring prospective interventional trials with appropriate patient selection, dose-ranging, and clinical endpoints before any clinical consideration. Frailty-stratified sepsis patients may be candidates for targeted cell death pathway interventions. Given the dominant pyroptotic signature in frail patients, NLRP3 inhibitors (e.g., MCC950, dapansutrile) support the investigation of approaches to attenuating gasdermin D cleavage and DAMP release in this population (Coll et al., 2015); prospective interventional trials are required before any clinical implementation. For patients with co-dominant necroptosis (high RIPK3), RIPK3 kinase inhibitors (GSK′872, receptor-interacting protein kinase inhibitors in phase I trials) provide biological plausibility for limiting endothelial necroptosis and organ crosstalk damage (M et al., 2014). The ferroptotic signature in frail patients supports the investigation of GPX4 stabilizers (ferrostatin-1, liproxstatin-1 analogs) or iron chelation strategies (Dixon et al., 2012). While none of these agents has established efficacy in clinical sepsis trials, our data provide a biological rationale for mFI-stratified biomarker enrichment in future trials. The present data provide biological plausibility for pathway-targeted approaches but constitute neither efficacy evidence nor a basis for treatment recommendations.

Several limitations should be acknowledged. The retrospective single-center design introduces potential selection and information bias. First, cell death biomarkers were measured at 48 h after sepsis onset—not preoperatively—and therefore cannot serve as prospective predictors of sepsis incidence. The biomarker findings have been reframed as prognostic (stratifying severity once sepsis is diagnosed) rather than predictive. A prospective study measuring biomarkers preoperatively, or at the earliest standardized postoperative time point prior to sepsis diagnosis, would be required to establish genuine predictive utility. Biomarkers were also measured at a single time point and do not capture the dynamic evolution of PCD pathways over the sepsis course; serial measurements at 24, 48, and 96 h post-onset would be required to characterize pathway kinetics. Second, the sepsis cohort (n = 30) is critically underpowered. The original 10-predictor logistic model yielded EPV = 3.0, far below the recommended minimum of 10; the model was accordingly reduced to three preoperative predictors (EPV = 10.0). Bootstrapped internal validation (1,000 iterations, optimism-corrected AUC = 0.704) reduces but cannot eliminate overfitting; external validation in an independent, adequately powered multicenter cohort is essential before any clinical claims are made. The small absolute number of sepsis patients (n = 30), particularly in the non-frail subgroup (n = 1), limits subgroup analyses and may affect the stability of mFI-stratified biomarker correlations. Biomarker measurements were not performed on all 188 patients, limiting a prospective biomarker-based sepsis prediction analysis; the matched control design mitigates but does not eliminate confounding. Finally, we did not assess tissue-level histological evidence of cell death, which would provide mechanistic validation of the circulating biomarker findings. Multicenter prospective studies with longitudinal biomarker profiling and pathological correlation are warranted.

In conclusion, this study demonstrates that mFI-defined frailty is a clinically accessible predictor of both post-THA infection-related sepsis incidence and the severity of concurrent PCD pathway activation. The frailty-cell death axis identified here—where higher mFI corresponds to amplified pyroptosis, necroptosis, ferroptosis, and DAMP release—offers a mechanistic framework for understanding why frail surgical patients disproportionately develop severe sepsis and organ dysfunction. A combined frailty-cell death biomarker model provides superior perioperative sepsis risk stratification and may ultimately guide patient selection for emerging cell death-targeted therapies in the rapidly expanding elderly THA population.

## Data Availability

The datasets for this article are not publicly available due to concerns regarding participant privacy. Requests to access the datasets should be directed to Minjie Yang (yangl2431@sina.com) and will be considered subject to institutional and ethics approval.
