# Supplementary material for: Modified frailty index predicts programmed cell death activation and sepsis risk after total hip arthroplasty: a retrospective cohort study
Source: Front Cell Dev Biol. 2026 Aug 21;14:1888879. doi: 10.3389/fcell.2026.1888879 (PMC13541678; doi:10.3389/fcell.2026.1888879)
Supplement: Supplementary file 1 [file Supplementaryfile1.docx]

Wei Y, Xu S, Yang M (2026) · Front. Cell Dev. Biol. · doi: 10.3389/fcell.2026.1888879

**Supplementary Table S1 |** Individual patient-level phospho-MLKL flow cytometry data in post-THA sepsis patients stratified by Modified Frailty Index (mFI). n = 30 (all patients who developed infection-related sepsis within 30 days of THA).

| **Patient ID** | **mFI Frailty Group** | **mFI Score** | **Age (years)** | **Sex** | **Sepsis Source** | **SOFA (72 h)** | **Phospho-MLKL Positivity (%)** | **Phospho-MLKL MFI (AU)** | **Total CD45+ Cells Gated** | **30-day Outcome** |
| --- | --- | --- | --- | --- | --- | --- | --- | --- | --- | --- |
| THA-S01 | Non-frail | 0 | 72 | M | SSI | 6 | 34.2 | 1,842 | 9,951 | Survived (ward) |
| THA-S02 | Mild frailty | 1 | 66 | M | UTI | 5 | **84.5** | 3,191 | 11,374 | Survived (ward) |
| THA-S03 | Mild frailty | 2 | 67 | M | UTI | 8 | 64.0 | 3,508 | 10,985 | Survived (ICU) |
| THA-S04 | Mild frailty | 2 | 68 | F | UTI | 9 | 68.0 | 3,335 | 9,546 | Survived (ICU) |
| THA-S05 | Mild frailty | 2 | 69 | M | UTI | 9 | 62.2 | 2,872 | 11,167 | Survived (ICU) |
| THA-S06 | Mild frailty | 2 | 71 | F | UTI | 5 | 64.3 | 2,728 | 11,082 | Survived (ward) |
| THA-S07 | Mild frailty | 2 | 71 | F | Bacteraemia | 4 | 50.5 | 2,622 | 12,056 | Survived (ward) |
| THA-S08 | Mild frailty | 2 | 71 | M | Pneumonia | 8 | 43.0 | 3,421 | 11,521 | Survived (ICU) |
| THA-S09 | Mild frailty | 2 | 73 | F | Pneumonia | 4 | 63.7 | 3,085 | 11,538 | Survived (ward) |
| THA-S10 | Mild frailty | 2 | 73 | F | Pneumonia | 8 | 26.4 | 4,215 | 10,297 | Survived (ICU) |
| THA-S11 | Mild frailty | 2 | 74 | M | Bacteraemia | 8 | 65.6 | 3,283 | 9,878 | Survived (ICU) |
| THA-S12 | Mild frailty | 2 | 76 | F | Bacteraemia | 6 | 62.6 | 4,268 | 11,569 | Survived (ward) |
| THA-S13 | Mild frailty | 2 | 76 | F | UTI | 9 | 46.7 | 2,594 | 9,965 | Survived (ICU) |
| THA-S14 | Mild frailty | 2 | 78 | F | UTI | 8 | 57.7 | 3,260 | 12,083 | Survived (ICU) |
| THA-S15 | Mild frailty | 3 | 79 | F | UTI | 9 | 44.3 | 2,513 | 10,778 | Survived (ICU) |
| THA-S16 | Mild frailty | 3 | 79 | F | UTI | 7 | 41.8 | 3,150 | 10,618 | Survived (ICU) |
| THA-S17 | Mild frailty | 3 | 79 | F | UTI | 4 | 68.6 | 3,725 | 11,904 | Survived (ward) |
| THA-S18 | Mild frailty | 3 | 80 | F | Bacteraemia | 7 | 57.8 | 3,062 | 10,211 | Survived (ICU) |
| THA-S19 | Mild frailty | 3 | 81 | F | SSI | 9 | **83.2** | 3,132 | 10,288 | Survived (ICU) |
| THA-S20 | Mod-severe frailty | 4 | 71 | F | Pneumonia | 8 | **94.8** | 5,301 | 12,087 | Survived (ICU) |
| THA-S21 | Mod-severe frailty | 4 | 72 | M | Pneumonia | 7 | **88.4** | 6,415 | 11,553 | Survived (ICU) |
| THA-S22 | Mod-severe frailty | 4 | 73 | M | Bacteraemia | 11 | **81.3** | 4,368 | 11,853 | Died (30-day) |
| THA-S23 | Mod-severe frailty | 4 | 73 | M | Bacteraemia | 13 | **94.8** | 4,261 | 11,765 | Died (30-day) |
| THA-S24 | Mod-severe frailty | 5 | 77 | M | Pneumonia | 13 | 44.0 | 4,564 | 12,251 | Died (30-day) |
| THA-S25 | Mod-severe frailty | 5 | 77 | M | Pneumonia | 8 | 54.2 | 4,929 | 9,582 | Survived (ICU) |
| THA-S26 | Mod-severe frailty | 5 | 81 | M | Pneumonia | 12 | **73.2** | 4,465 | 12,137 | Died (30-day) |
| THA-S27 | Mod-severe frailty | 6 | 83 | M | SSI | 11 | **83.0** | 5,233 | 10,882 | Died (30-day) |
| THA-S28 | Mod-severe frailty | 7 | 84 | M | Bacteraemia | 10 | **78.8** | 5,578 | 11,167 | Survived (ICU) |
| THA-S29 | Mod-severe frailty | 7 | 86 | M | SSI | 10 | **79.1** | 4,135 | 12,244 | Survived (ICU) |
| THA-S30 | Mod-severe frailty | 7 | 87 | F | SSI | 8 | **90.7** | 6,199 | 12,443 | Survived (ICU) |

**Abbreviations:** mFI, modified frailty index; MFI, median fluorescence intensity; AU, arbitrary units; SOFA, Sequential Organ Failure Assessment; THA, total hip arthroplasty; SSI, surgical site infection; UTI, urinary tract infection; MLKL, mixed lineage kinase domain-like protein; CD45, leukocyte common antigen.

**Notes:** Phospho-MLKL positivity (%) = percentage of CD45+ peripheral blood mononuclear cells with detectable phospho-MLKL signal above isotype control threshold (~1.5%), measured by intracellular flow cytometry (Cell Signaling Technology #37705, Alexa Fluor 647 secondary antibody) at 48 h post-sepsis onset. MFI values are normalized to the isotype control median. Bold red values (≥70%) indicate markedly elevated phospho-MLKL positivity. Outcome: Survived (ward) = no ICU required; Survived (ICU) = ICU admission but alive at 30 days; Died (30-day) = death within 30 days of sepsis onset (highlighted in red). Non-frail subgroup (n = 1): descriptive only; no between-group inference applicable. Group summary — Non-frail: 34.2% (n=1); Mild frailty: 58.6 ± 14.2% (mean ± SD, n=18); Moderate-to-severe frailty: 78.4 ± 18.6% (n=11); trend P = 0.003 (Jonckheere–Terpstra test).

**Supplementary Figure S1 | Calibration Plot for the Preoperative Prediction Model for Post-THA Infection-Related Sepsis.**

**Figure S1.** Calibration plot for the preoperative logistic regression model predicting post-THA infection-related sepsis. The x-axis shows mean predicted sepsis probability within each decile of predicted risk; the y-axis shows the observed proportion of patients who developed sepsis. Circles denote decile midpoints; error bars represent 95% Wilson confidence intervals for observed proportions. The dashed diagonal line indicates perfect calibration (observed = predicted). The smoothed calibration curve (blue) is a cubic-spline fit. D1–D10 = decile 1 to 10 of predicted risk; n = patients per decile (≈18–19). The preoperative model comprised three predictors: mFI score, preoperative haemoglobin, and ASA classification (events-per-variable = 10.0). Adequate calibration was confirmed by Hosmer–Lemeshow goodness-of-fit test (χ² = 7.84, df = 8, P = 0.449). Bootstrap internal validation (1,000 iterations) yielded an optimism-corrected AUC of 0.704 from an apparent AUC of 0.742. External multicenter validation is required before clinical implementation.

**Statistical note:** Calibration was evaluated across 10 equal-frequency deciles of predicted risk (n = 188 total; 30 events; ≈18–19 patients per decile). Predicted probabilities span 2.2% (lowest decile) to 41.8% (highest decile). The lack of systematic over- or under-prediction (P = 0.449 on Hosmer–Lemeshow test) indicates adequate model calibration across the observed risk range. Note that the optimism-corrected AUC (0.704) represents the more conservative and generalizable performance estimate.

**Abbreviations:** mFI, modified frailty index; THA, total hip arthroplasty; ASA, American Society of Anesthesiologists; AUC, area under the ROC curve; CI, confidence interval; EPV, events-per-variable.
